# Insight into the Electrical Double Layer of an Ionic Liquid on Graphene

**DOI:** 10.1038/s41598-017-04576-x

**Published:** 2017-06-26

**Authors:** L. Andres Jurado, Rosa M. Espinosa-Marzal

**Affiliations:** University of Illinois at Urbana-Champaign, Urbana, USA

## Abstract

Graphene is a promising next-generation conducting material with the potential to replace traditional electrode materials in supercapacitors. Since energy storage in supercapacitors relies on the electrolyte-electrode interface, here we elucidate the interfacial subnanometer structure of a single component liquid composed solely of cations and anions – an ionic liquid- on electrified graphene. We study the effect of applied potential on the interaction between graphene and a silicon tip in an ionic liquid and describe it within the framework of the Derjaguin-Landau-Verwey-Overbeck (DLVO) theory. The energy is stored in an electrical double layer composed of an extended Stern layer, which consists of multiple ion layers over ~2 nanometers, beyond which a diffuse layer forms to compensate the applied potential on graphene. The electrical double layer significantly responds to the applied potential, and it shows the transition from overscreening to crowding of counterions at the interface at the highest applied potentials. It is proposed that surface charging occurs through the adsorption of the imidazolium cation to unbiased graphene (likely due to π-π interactions) a﻿nd that the surface potential is better compensated when counterion ﻿crowding happens. This study scrutinizes the electrified graphene-ionic liquid interface, with implications not only in the field of energy storage, but also in lubrication.

## Introduction

Since its discovery in 2004^1^, graphene has revolutionized a new field of study in 2D nanomaterials^2^. Made up of a honeycomb carbon lattice, it has been found to exhibit many unique properties including ballistic electron transport^3^, large in-plane elastic modulus^4^, and high thermal conductivity^5^. Graphene owes its superior electronic properties to its honeycomb lattice carbon network, in which 2s, 2px, and 2py orbitals hybridize such that each carbon atom is bonded to its three neighbors by strong “sigma” bonds. The remaining “π” orbital determines the low-energy electronic structure of graphene. These desirable properties make graphene of considerable interest for potential use in many applications, several of which rely on liquid-graphene interfacial properties, including its use as supercapacitor electrode^6–8^. Interfacial water consisting of monolayers and multilayers have been often observed between graphene films and hydrophilic substrates such as mica and sapphire^9, 10^, and between graphene sheets^11^. A fundamental understanding of the interfacial liquid properties imposed by electrified graphene is, however, still lacking.

A single component electrolyte composed of ions –an ionic liquid– with a moderate polarizability has been chosen for this study. Ionic liquids (ILs) are organic molten salts with low melting point^12^; due to its ionic composition, they are subjected to significant electrostatic interactions. Because of their high charge density and wide electrochemical window, and the possibility to tune intermolecular forces and physicochemical properties, ILs are considered to be ideal electrolytes for energy storage^13^. ILs display more pronounced ordering than conventional electrolytes due to strong ion-ion interactions -especially of Coulombic, van der Waals and solvophobic origin-, and many form well-defined nanostructures in the bulk phase^14^, at solid surfaces^15^ and in confinement^16^. Experiments^17, 18^ show that ILs near the solid surface commonly exhibit oscillatory density profiles, which reflect the layered arrangement of the IL ions at the buried liquid-solid interface. Besides out-of-plane order, in-plane order has been also demonstrated by atomic force microscopy and scanning tunneling microscopy.^19^ Several parameters have been found to influence the interfacial structure, such as the chemical composition of the surface^20^, the strength of ion-ion interactions, *i.e*. the IL molecular structure^21^, and the surface charge and potential^22, 23^. Increasing surface potential leads to an increase in the strength of the ion-surface Coulombic interaction.^24^ Further, theoretical studies have shown that, when the electrode polarization increases, the interfacial structure of ILs undergoes a transition from *overscreening* the surface charge by a single monolayer of counterions to *crowding* of counterions across more than a single monolayer^25^.

Over the past three years some evidence has accumulated demonstrating that long range surface forces on electrically charged surfaces immersed in ILs are of electrostatic origin and they respond to the characteristics of an electrical double layer force of a dilute electrolyte solution^26^. A long-range electrical double layer force with a decay length as large as 13 nm has been measured for several ILs on various substrates^26–31^. This finding is important since the interfacial capacitance is inversely proportional to the effective thickness of the electrical double layer where the charge is stored, and hence, it affects the stored energy in the supercapacitor. The proposed “aggregation” model^26^ assumes that ILs are dielectric solvents composed of a strongly correlated ionic network, where each charge is counterbalanced by the sum of the neighbours’ charge and only a small percentage of the ionic network is effectively dissociated. To reconcile the aggregation model with NMR diffusivity measurements, which fail to find evidence for long lived ion pairs^32^, the coordinated cation-anion network is proposed to be highly dynamic and transient. Although the temperature-dependence of the electrical double layer force further strengthens the argument in favor of a diffuse layer, the shape of the reported capacitance and ion conductivity in separate works and in different systems contradicts the assumption of low ion dissociation, and it is still a subject of debate^13, 26^. Nevertheless, our results are consistent with the reported aggregation model.

To date, a few simulations, scattering and spectroscopy works have investigated the IL-graphene interface^33–38^. The results are contradictory with regard to the composition of interfacial IL layers, since some of them show a significant enrichment of imidazolium cations on uncharged graphene^34, 36^, while others show a densification of the interfacial layer composed of ion pairs, although, admittedly, π-π interactions are missing in these simulations^38^. A thorough experimental study of the Stern and diffuse layers of ILs on electrified graphene has not been performed yet. In this work, we have scrutinized the electrical double layer of 1-ethyl-3-methylimidazolium bis(trifluoromethylsulfonyl)imide (abbreviated as [EMIM][TFSI]) at the graphene plane, for single- and few-layer graphene by probing normal surface forces by atomic force microscopy under applied positive and negative potentials. Statistical analysis of the steps resolved in the force-separation curves and modeling of the surface forces provide molecular insight into the electrical double layer of [EMIM][TFSI].

## Results

A 90 nm thick silicon dioxide layer –that we refer as silica substrate– and a working electrode composed of a 9 nm thick gold layer thermally evaporated on the silica substrate were chosen as substrates for mechanically exfoliated graphene. The number of mechanically-exfoliated graphene sheets was determined by combining Raman microspectroscopy and atomic force microscopy (AFM) imaging of the topography. All graphene samples were first imaged in air and then in [EMIM][TFSI] to exclude changes of topography upon immersion in the IL. Figure S1 in Supplementary Material (SM) shows AFM images and Raman spectra for one of the samples. More experimental details can be found in Materials and Methods.

### Normal force-separation curves

**Figure 1:**
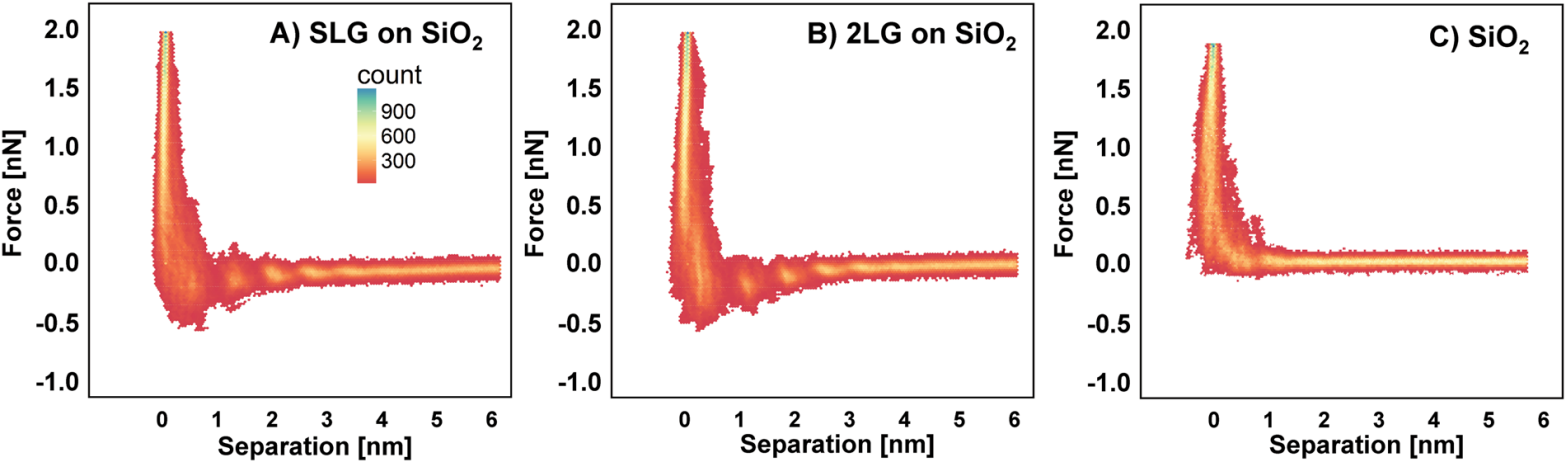
Bivariate histograms for the normal force as a function of the separation (with arbitrary zero at the hard wall at 10 nN) for SLG (**A**), 2LG (**B**), and silica substrate (**C**). FLG is in the SM (Figure S2). The bivariate histograms for the force-separation curves were constructed via hexagonal binning, with a bin size of 150. Red highlights regions of low data density, whereas yellow regions highlight regions of high data density. Due to the inherent uncertainty of the absolute tip-substrate separation in AFM force measurements, we note that the abscissa has an arbitrary zero but we label it as “separation”. Figure S1 shows AFM images of the graphene sample and the corresponding Raman spectra. Spring constant of cantilever = 0.3507 N/m and Si-tip with radius = 35 nm.

After equilibration, normal force-separation curves were measured on edge-free areas of single-layer (SLG), bilayer (2LG), few-layer (FLG, 3–4 layers), and multi-layer (MLG) ( > 7) graphene supported on gold and silica at a constant approach speed of 10 nm/s. Figure 1(A–C) shows bivariate histograms of force–separation curves in [EMIM][TFSI] collected on silica-supported single-layer and bilayer graphene sheets, as well as on the underlying silica support, all measured with a silicon tip; Figure S2 in SM shows similar results for FLG on the same silica substrate. At least 64 force measurements were superposed in each diagram. It is evident that an attractive force of similar magnitude is measured for graphene independently of the number of graphene sheets, thereby diverging from the weakly repulsive force on silica. Measurements on other samples are qualitatively similar (Figure S3). The negligible influence of the substrate beneath graphene on the attractive force is further confirmed by comparing to the force measurements on gold-supported graphene (Figure S4).

Discontinuities in all force-separation curves appear at separations smaller than D ~ 2–3 nm superposed to an attractive force; the “red” regions within the clouds of data points with higher density represent these discontinuities in a bivariate histogram. The discontinuities, which are referred to as film-thickness-transitions (FTT), reflect the oscillatory density profile of liquids at interfaces^39, 40^, i.e. the layered arrangement of the molecules at the buried liquid-graphene interface. The FTT happens when the force required to overcome a maximum in the oscillatory force is reached. In terms of mechanics, if the second derivative of the effective surface interaction potential exceeds the spring constant of the cantilever, the tip will push through an IL layer, thereby leading to a step in the force-separation curve. On unbiased graphene, ~4–7 FTTs are resolved in each force-separation curve, i.e. more than on silica and gold supports (~3–4). While the atomically smooth graphene plane is expected to favor efficient ion packing at the interface compared to silica, the roughness of gold is also sub-nanometer (RMS ~0.3 nm as determined by AFM), and hence, only surface roughness cannot explain the enhanced IL-layering on graphene.

**Figure 2:**
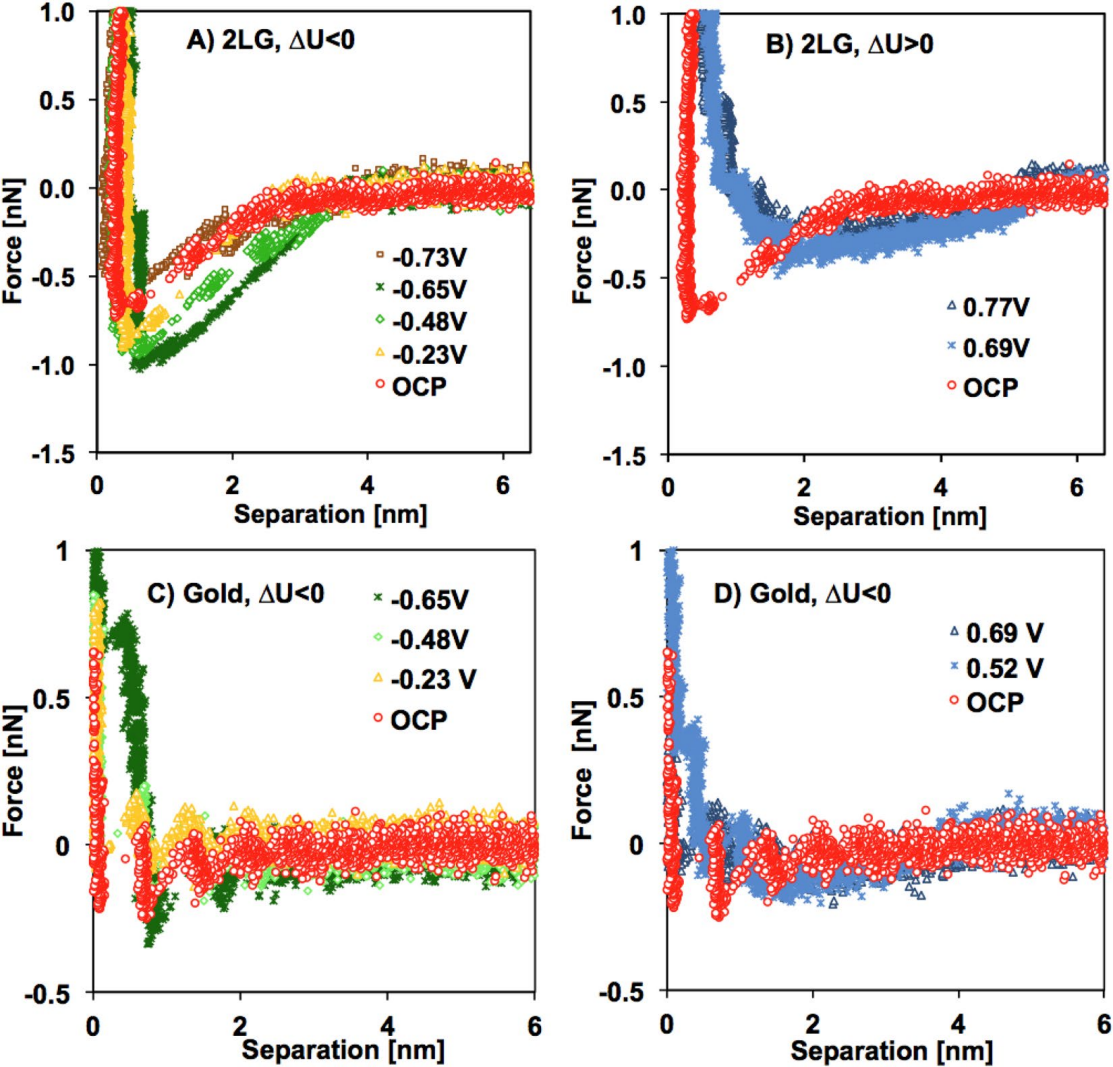
Selected force-separation curves for gold-supported bilayer graphene (**A**,**B**) and gold (**C,D**) at negative (**A**,**C**) and positive (**B**,**D**) potentials measured with an AFM tip. Spring constant of cantilever = 0.375 N/m and silicon tip with radius = 20 nm. Note the different minimum value of the Y-axis.

Since the number of graphene sheets did not significantly influence the interaction between tip and supported graphene in the IL (Figure S4), the investigations under applied potential were limited to bilayer graphene supported on gold. A home-made three-electrode-cell attached to the AFM stage with platinum wires as counter- and reference electrodes was used for these experiments; for more details, see Materials and Methods. Potentials were applied to the gold substrate, taking into consideration that the electrochemical window of [EMIM][TFSI] is ~2 V (Figure S5). Figures S6 and S7 show the bivariate histograms for the force-separation curves as a function of the applied potential measured on bilayer graphene and on the gold support, respectively. Figure 2A,B and C,D display representative force-separation curves on bilayer graphene and gold, respectively, to facilitate comparison at the selected potentials.

On graphene (Fig. 2A,B), the force-separation curves remarkably respond to changes in applied potential, and although the force remains negative at positive (∆U > 0) and negative (∆U < 0) potential, i.e. attractive, the onset notably increases from ~4 nm at open circuit potential (OCP) up to ~6 nm. By decreasing the negative potential (−0.23, −0.48 V and −0.65 V), the force becomes more attractive, while the trend reverses by further decreasing the potential to −0.73 V. Similarly, Figure S8 in SM shows the same trends at positive and negative potentials for another graphene sample. We note that the attractive surface forces between the tip and graphene are consistent with reported measurements on HOPG for the same IL^21^. This previous work also detected an attractive force but did not rationalize its origin. We propose that the electrical double layer (EDL) force is attractive for the dissimilar graphene-tip system and that the variation of the force with surface potential reflects the change of the EDL with applied potential. Further, the decrease in the attractive EDL force at the highest applied potentials(~ ± 0.7 V) is related to the onset of crowding, as discussed later.

The difference between the normal force on gold and bilayer graphene at all potentials is remarkable. The force is much less attractive on gold than on graphene. Thus, either the applied potential on gold is better compensated by the strongly adsorbed IL counterions (as proposed previously when comparing mica and gold^28^), or the Hamaker constant on gold is much smaller than on graphene as a result of the unique electronic properties of graphene; the discussion of the Hamaker constant in the next section does not support the latter, though. Nevertheless, the consistently lesser sensitivity of the measured force on gold to surface potentials compared to graphene (see also Figure S7) emphasizes the distinct EDL of [EMIM][TFSI] on gold and graphene, as discussed later.

### Pull-off force and electrowetting

**Figure 3:**
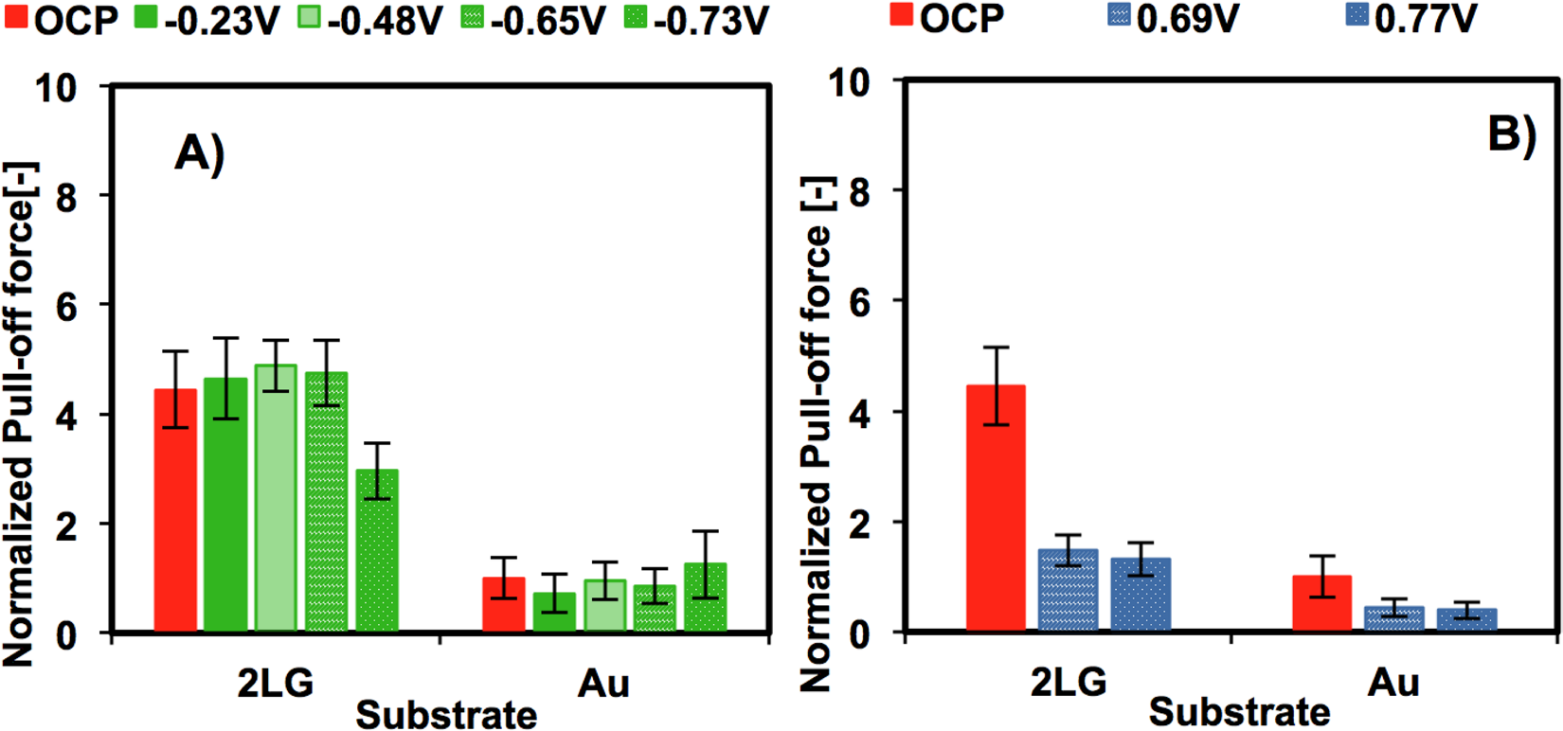
Pull-off force between the silicon tip and either bilayer graphene (2LG) or the gold substrate normalized by the pull-off force on gold at OCP (~0.7±0.3 nN): (**A**) OCP and negative potentials (∆U < 0); (**B**) OCP and positive potentials (∆U > 0).

The force-separation curve upon retraction, specifically the pull-off force, gives information about the interfacial energy, which includes the contribution of van der Waals, oscillatory entropic solvation and electrostatic forces^39^. Figure 3 shows a summary of the pull-off force measured on bilayer graphene and on gold in [EMIM][TFSI] normalized by the pull-off force on gold at OCP (~0.7±0.3 nN) as a function of the applied potential. The pull-off force on graphene is ~3–4 times larger than on gold and ~7–9 times larger than on silica (∼0.33±0.1 nN for silica, Figure S9A).

Since the Hamaker constant of gold (a metal) is expected to be larger than that of graphene, the higher pull-off force on graphene in the absence of an applied potential (OCP) might appear surprising. However, due to the presence of oscillatory entropic solvation forces, multiple adhesive minima are present in confined liquids that arrange in layers. Such adhesive minima depend on the number of confined layers, as already demonstrated for ILs. ^41^. Since the absolute separation is unknown in AFM force measurements, it is possible that the pull-off force was measured at different adhesive minima on gold and graphene at OCP.

The pull-off force remarkably decreases when the potential goes beyond a threshold (~−0.6 V at negative potentials. The significant decrease in adhesion with potential reflects the electrowetting of graphene by [EMIM][TFSI] induced by the applied potential, which implies that the surface potential modifies the EDL. An increase in stored energy in the electrical double layer is responsible for the decrease in adhesion energy^42^. Figure S9B shows that the potential threshold as well as the pull-off force is smaller at positive potentials (~0.2 V). The electrowetting phenomenon is thus asymmetric, likely because of the different molecular composition of anion and cation and their different interactions with the surface^43^. Asymmetric electrowetting has been also measured for deep eutectic solvents composed of choline chloride in organic solvents like urea, ethylene glycol and glycerol on HOPG, and hence, it is not a unique feature of ILs^44^.

### Derjaguin-Landau-Verwey-Overbeck (DLVO) theory

To get more insight into the EDL of [EMIM][TFSI] on graphene, the measured surface forces were modeled according to the DLVO theory. The following expression gives the DLVO force for dissimilar surfaces as the sum of van der Waals and electric double layer forces^28^:

1 $$\frac{{{\rm{F}}}_{{\rm{DLVO}}}}{{\rm{R}}}=-\frac{{{\rm{A}}}_{{\rm{G}}}}{6{{\rm{D}}}^{2}}+\frac{2{\rm{\pi }}{\rm{\kappa }}\epsilon {\epsilon }_{0}(2{{\rm{\psi }}}_{{\rm{G}}}{{\rm{\psi }}}_{{\rm{tip}}}-({{\rm{\psi }}}_{{\rm{G}}}^{2}+{{\rm{\psi }}}_{{\rm{tip}}}^{2})\exp (-{\rm{\kappa }}{\rm{D}}))}{(\exp ({\rm{\kappa }}{\rm{D}})-\exp (-{\rm{\kappa }}{\rm{D}}))}$$

where R is the AFM probe radius (in m), A_G_ is the Hamaker constant (in J), D is the surface separation (in m), ψ_G_ is the potential at the Outer Helmholtz Plane (OHP) on graphene (in V), ${{\rm{\psi }}}_{tip}$ is the diffuse potential of the (naturally oxidized) silicon tip (in V), κ is the inverse of the Debye length (in m^−1^), ε is the relative permittivity of the IL (unitless), and ε_o_ is the vacuum permittivity (in F/m). The expression for the EDL force is based on Hogg-Healy-Fuerstenau approximation^45^ and it assumes constant surface potential as boundary condition; neither constant surface charge nor mixed boundary conditions were found to agree with the experimental data. The second term in the EDL force always leads to an attractive force, whereas the first term can cause an attractive or a repulsive force depending on the sign of the diffuse potentials. Calculations were not carried out for DLVO forces between silica or gold supports and the tip due to the small value of the measured surface forces at D > 2 nm.

The van der Waals term in Eq. (1) assumes no transparency of graphene to dispersion interactions between the tip and the underlying metallic or dielectric substrate^46, 47^. The Lipkin theory^48^ was used to estimate the Hamaker constant of graphene and tip (considered to be naturally oxidized silicon, i.e. SiO_2_) in air with ${n}_{Si{O}_{2}}$=1.47, ${\nu }_{e,Si{O}_{2}}$=3.2·10^15^ s^−1^, and $\,{\nu }_{e,G}$=4·10^15^ s^−1 46^, which yields A_G_ ~ 1.52·10^−19^ J and is close to reported experimental and theoretical values for HOPG (1.35·10^−19^ J in ref. 49); the same theory yields A_Au_ ~ 1.91·10^−19^ J for gold (${\nu }_{e,Au}$=6.2·10^15^ s^−1^). There is no agreement about the influence of the number of graphene sheets on the Hamaker constant yet^46, 49, 50^, but these results suggest the influence to be small. According to mixing rules, the IL is expected to reduce the Hamaker constant with respect to the values obtained in air^39^. Gebbie *et al*.^28^ argued that surface-adsorbed IL ions can further affect the Hamaker constant and used a value of ~0.9–2·10^−20^ J for mica-IL-gold systems. An accurate analytical estimation of the Hamaker constant for the AFM probe-IL-graphene system is not possible yet. Satisfactory fits to the force-separation curves with Eq. (1) were achieved with A_G_ ~ 1.1·10^−20^ J, which was estimated according to mixing rules with ${n}_{IL}$=1.412, ${{\epsilon }}_{IL}$=12 and ${{\epsilon }}_{Si{O}_{2}}$=3.8.

**Figure 4:**
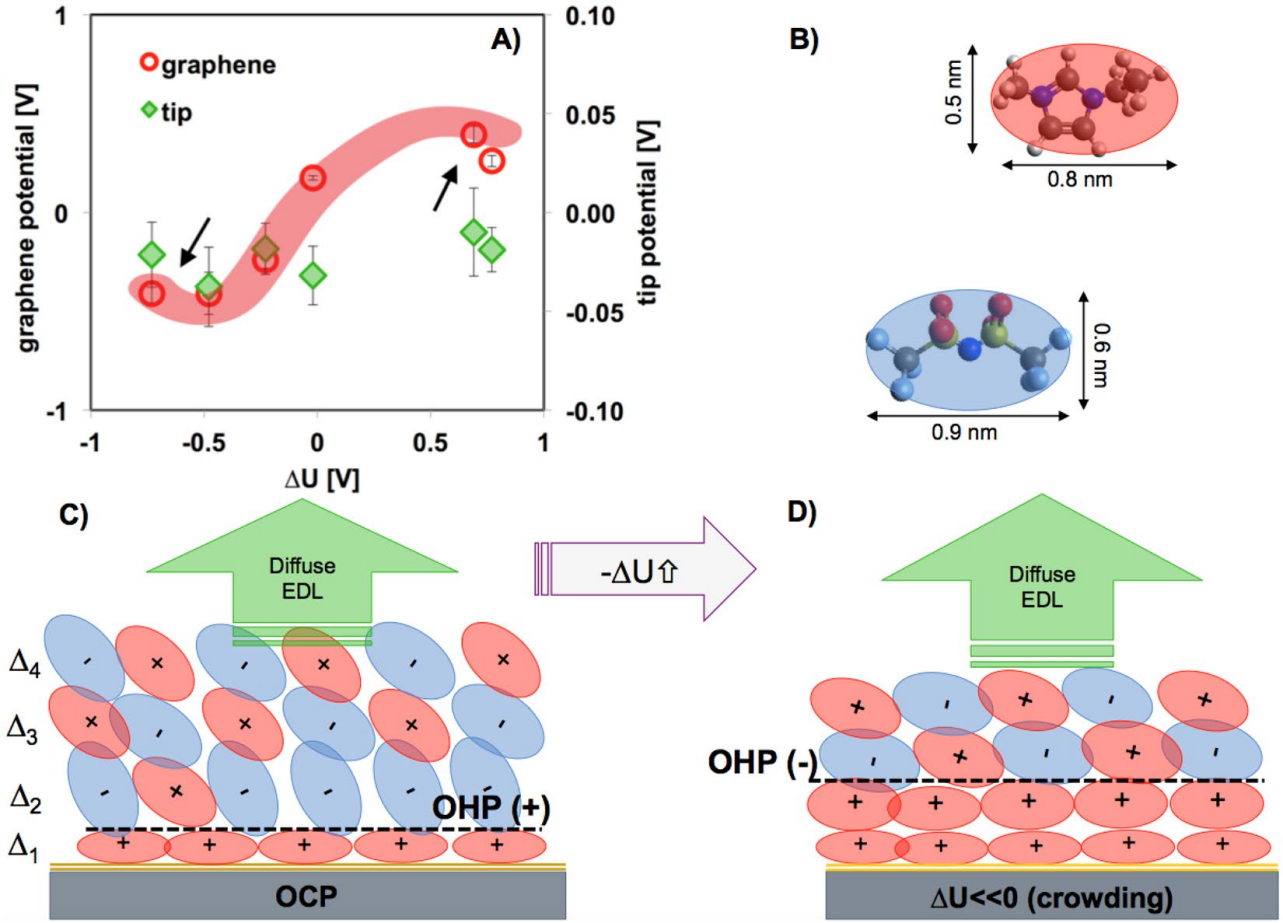
Proposed model for the EDL of [EMIM][TFSI] on graphene electrodes as a function of the potential. (**A**) The diagram shows the effective diffuse potentials on graphene and on the AFM tip. (**B**) Dim**e**nsions of cation and anion in the absence of nanoconfinement as calculated by the Avogadro software v.1.1.1. Proposed illustration of the EDL of [EMIM][TFSI] at OCP (**C**), and after the onset of crowding (**D**). The illustrations assume that the hard wall is at the graphene plane, and hence, it neglects a surface-bound layers of counterions that the AFM tip might not be able to remove at the highest load. A further simplification of the cartoon concerns the innermost layer at OCP, which might be cation rich instead of composed solely of cations. The accurate position of the OHP is also unknown.

Since Eq. (1) does not account for short-range non-DLVO forces such as solvation forces, the DLVO force was fitted to all force-separation curves at separations D > 2 nm assuming a constant decay length of 6.6 nm (${\kappa }^{-1}=0.15$ nm^−1^), as previously determined for [EMIM][TFSI] in the potential range + /− 0.5 V)^27^. The good fit of the DLVO theory to the measured surface forces under applied potential demonstrates that an EDL force can explain the change in the measured attractive interaction (see an example in Figure S10). Thus, the *effective* diffuse potentials for tip and graphene (${\psi }_{tip}$ and ${\psi }_{G}$) were obtained within the context of the classical Poisson-Boltzman theory according to Eq. (1). Figure 4A shows a summary of the obtained fitting parameters, ${\psi }_{tip}$ and ${\psi }_{G}$. It is to be noted that the normalized force (F/R) in Eq. (1) only describes a surface force under the condition R ≫ D according to the Derjaguin approximation. In our experiments, R is ~20–50 nm, and hence, this approximation is not strictly valid. However, previous work has demonstrated the very good agreement between theory and force-separation curves with a similar sharp tip^51^, and hence, the results are expected to be accurate.

The effective diffuse potential of the tip is small and negative at all applied potentials (${\psi }_{tip}$=−23 ± 10 mV). For graphene, ${\psi }_{G}$ is positive (~0.17±0.01 V). We note that a layer composed of [EMIM]^+^ cations with the ring lying parallel to the surface would yield ~1.6 positive charges per nm^2^. In comparison, the negative charge density of mica is ~ −2 nm^−2^, while a surface potential as high as −0.48 V has been reported for fully ionization of mica in water. Hence, a value of + 0.17 V is consistent with the presence of a surface-adsorbed IL-layer greatly enriched in cations.

For increasing negative potentials (ΔU < 0), the stronger attractive force leads to a more negative effective diffuse potential on graphene (Fig. 4A). A better compensation of the applied potential is observed at the highest negative potential (−0.73 V), when the fitted potential achieves a plateau (see arrow). At positive potentials (∆U > 0), the fitted potential on graphene, ${\psi }_{G}$, is positive, but the trends are similar to those observed at negative potentials.

Figure S11 points at force-separation curves, in which an instability was detected at the negative potential of −0.73 V, at which the force-separation curve suddenly became more attractive, which suggests lesser compensation of the applied potential. The force remained strongly attractive after reversing the potential to positive values; beyond a positive potential (here 0.52 V), the force became eventually less attractive. Such instability was never observed on gold, and only sometimes on graphene; Figs 2 and S8 show results in the absence of this instability. The instability in the force-separation curve was also reflected in a significant increase in pull-off force (up to ~6.4 nN), which indicates that the energy stored within the EDL significantly decreased under these conditions. Although the origin of this instability is still unknown, we report it here because the implications for energy storage could be important.

### Out-of-plane structure of the “extended” Stern layer

The structure of the quantized region of the EDL –we call it the *extended* Stern layer because it is composed of multiple layers– can be determined through statistically scrutiny of the film-thickness transitions detected in the force-separation curves. The size of a film-thickness transition is defined as ${\rm{\Delta }}={D}_{2}-{D}_{1}$, where ${D}_{2}$ and ${D}_{1}$ are the surface separation before and after a layer has been pushed through with the AFM probe, respectively. The change in film thickness, ∆, is commonly referred as the layer thickness. It is to be noted that the measurements on different samples were performed with different tips and its radius could be different, thereby influencing the magnitude of the force required to push through a layer, but not the ∆-values that are discussed here^52^. 2D histograms with the layer size (∆) and the layering force (F) were constructed to compare the composition of the four IL-layers closer to the hard wall on graphene, and silica and gold supports (Figures S12–S14).

**Figure 5:**
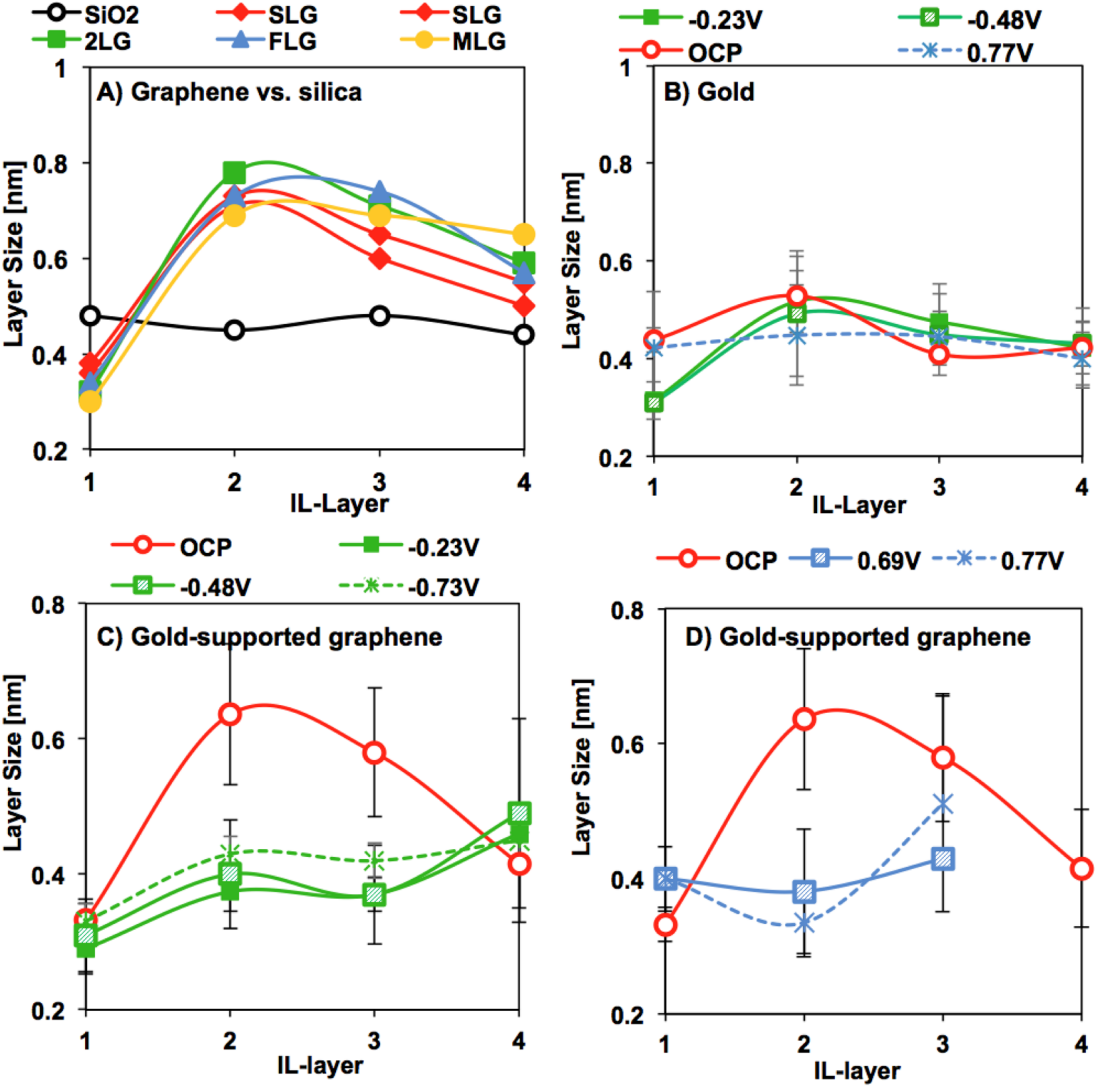
Structure of the *extended* Stern layer on graphene, and gold and silica supports. The data points give the mean value of the Gaussian distribution fitted to the layer thickness distribution and the variance is shown as an error bar. For (**A**) silica and silica-supported graphene, (**B**) gold, and gold-supported bilayer graphene at (**C**) negative and (**D**) positive potentials.

The average IL-layer thickness and variance at the peak with highest frequency were determined by fitting multi-peak Gaussian distributions to the 2D histograms. Figure 5A shows the discrepant interfacial structure of [EMIM][TFSI] on silica and graphene (with various numbers of sheets). On the silica support, the thickness of the four detected IL-layers is ~ 4.8 Å. Assuming that ion pairs are hard spheres, the diameter of an [EMIM][TFSI] ion pair is ~9 Å, i.e. about twice the measured size of an IL-layer. In contrast, the layer size for [EMIM][FAP] on gold was reported to be approximately equal to the diameter of the ion pair^53^. There are two possible explanations for the small size of the layers of [EMIM][TFSI] on silica surfaces. First, more disordered layers appear to be smaller^54^; the surface roughness of silica and larger distances to the surface could be responsible for such disorder. Second, MD simulations have shown that the density of nanoconfined 1-methyl-3-methylimidazolium [MMIM][TFSI] remarkably increases above the values in the bulk and the IL layers –composed of ion pairs- have a ∆-value of ~ 4 Å when confined between neutral surfaces^55^. It is thus possible that the [TFSI]^-^ anion enhances the compressibility of the IL compared to the [FAP]^-^ anion, thereby justifying the small size of the layers resolved with the AFM probe upon compression. Based on this discussion, it is likely that IL-layers with ∆ ~ 4.8 Å are rich in ion pairs.

The thickness of the IL-layers on silica-supported graphene significantly deviates from the values measured on the silica support (Fig. 5A, Figure S12): the IL-layer closest to the hard wall is the smallest one (∆_1_ ~ 3.3 Å); the second and third IL-layers are much larger than on the silica support (∆_2_ ~ 7–8 Å, ∆_3_ ~ 6–7 Å), while the thickness of the following IL-layers decreases toward ∆ ~ 5 Å. The cartoon in Fig. 4C shows the proposed simplified structure of the *extended* Stern layer of [EMIM][TFSI] on graphene, consisting of a film with 4–7 IL-layers. The characteristic size of the IL-layer at the graphene plane (~3.3 Å) is consistent with a cation-enriched layer, in which the imidazolium ring is parallel to the graphene plane. A cation-enriched adlayer would yield a plane of net positive charge on unbiased graphene with a maximum overscreening of ~1.6 positive charges per nm^2^ (for an interfacial layer composed solely of [EMIM]^+^ cations), qualitatively consistent with the positive diffuse potential at OCP (Fig. 4A). Figure 4C shows the surface-adsorbed layer to be composed solely of cations, although it might be cation-rich instead. As a result of cation-enrichment and significant overscreening, anion-enrichment is favored in the 2^nd^ layer, which coincides with the expansion of this layer to accommodate the anions oriented perpendicular to the surface; similar orientation was observed for the anion [TFSI]^-^ in [MMIM][TFSI] in simulations^55^. Accordingly, a cation enrichment would be favored in the 3^rd^ layer, whereas the concentration of ion pairs is expected to increase gradually with the distance from the surface, which is consistent with the observed plateau in ∆ at ~5 Å for the 5^th^ and 6^th^ IL-layers (not shown).

We note that similar layer thicknesses (∆_1_ ~ 3.1 Å, ∆_2_ ~ 8 Å and ∆_3_ ~ 5 Å) were reported for [EMIM][TFSI] on HOPG (see Fig. 3a in ref. 21), thus, in good agreement with our results. In contrast, MD simulations and AFM force-spectroscopy showed a layer thickness of ∆ ~ 7 Å for [EMIM][TFSI] on a HOPG surface at zero charge^33^, which was attributed to a densely packed cation-anion layer. These seemingly contradictory results highlight the different surface properties of the materials used in each of these experiment, perhaps due to different sample preparation and storage conditions, and hence, degree of oxidation^50^. This is an important question that we aim to clarify in future.

We note that the extended Stern layers built up on gold- and silica-supported graphene are qualitatively similar (Fig. 5A vs. 5B,C), but the expansion of the second and third IL-layers is often less pronounced on gold-supported graphene. This difference in the interfacial structure may rely on the amount of trace water. In fact, simulations have shown that small amounts of water absorbed from ambient air are able to modify the layered structure of the confined IL^56^, also between graphene sheets^57^. The amount of trace water is expected to be higher in the experiments with silica-supported graphene compared to gold-supported graphene (see Materials and Methods). Since water molecules preferentially interact with the anion, they might be enriched in the second layer, where the highest expansion was observed on silica-supported graphene.

The applied potential greatly affects the layer size distribution of [EMIM][TFSI] on graphene (Fig. 5C,D). The size of the 1^st^ layer remains essentially unchanged at negative potentials, which is consistent with a cation-rich composition. The size of the second and third IL-layers responds to the applied negative potential through a smaller expansion, likely to result from the increase in near-surface cation population compared to OCP (Fig. 5C). At applied positive potentials, the Coulombic interactions between the anions and graphene also cause the expansion in the second layer to vanish. Reproducibly, the size of the 4^th^ layer increases, which might reflect that co-ion and ion pair enrichment is shifted further away from the surface with applied potential. Importantly, the number of IL layers decreases at positive potentials, at which at most 3 layers are detected, while the force required to push through these layers increases (see Fig. 2B). It is possible that the tip cannot push through a surface-bound IL layer at the highest applied load of 10 nN at positive potentials. Alternatively, the decrease in layering may be attributed to the higher configurational entropy of the [TFSI]^-^ anion compared to the cation and its lesser packing efficiency^58^.

The out-of-plane structure of [EMIM][TFSI] on gold is clearly different at all potentials. Figure 5B shows a slightly oscillatory layer size distribution at OCP (∆_1_ ~ 4.2 Å and ∆_2_ ~ 5.3 Å, ∆_3_ = ∆_4_ = 4.0 Å) that differs from the characteristic layer size distribution on silica and graphene surfaces. At positive potentials, the thickness of the 1^st^ layer does not change, which would be consistent with an anion-enrichment at OCP and ∆U > 0. The decrease in size of the 1^st^ layer to ∆_1_ ~ 3–3.5 Å at negative potentials clearly supports cation-enrichment and parallel orientation of the imidazolium ring relative to the surface (*6*), as observed for graphene. We note that previous work has shown the specific adsorption of 1-butyl-1-methylpyrrolidinium cations to gold at OCP and even at positive potentials^56^. However, the interaction between gold and [EMIM]^+^ is known to be weaker than between gold and 1-butyl-1-methylpyrrolidinium, and hence, this is unlikely to happen here^28, 58^. The observed increase in the layering force required to push through the IL layer closest to the hard wall under applied potential compared to OCP supports the last resolved layer to be enriched in counterions. The force differs under negative (∆F ~ 0.75–1 nN) and positive (∆F ~ 0.44 nN) potentials, which indicates the different binding strength of the (positively and negatively charged) counterions, justified by the ion pair asymmetry^59^ and in agreement with other works^23, 28^. Although no layers were resolved at loads above 2 nN, it cannot be totally excluded that IL remains confined between the AFM probe and the gold substrate at the maximum applied load. Such an effect could explain the smaller pull-off force on gold compared to graphene. Future studies, e.g. with our Surface Forces Apparatus, are needed to test this hypothesis on gold surfaces.

## Discussion

The described force measurements provide insight into the EDL of [EMIM][TFSI] on graphene. While EDL forces have been previously measured between charged or biased surfaces in ILs^26^, ion pairs are expected to populate the solid-liquid interface in the absence of surface charge and specific interactions, thereby eliminating overscreening and EDL forces, as it occurs on silica surfaces (Fig. 1C). However, the preferential adsorption of imidazolium cations onto unbiased graphene –also detected by spectroscopy and in simulations^34, 36^- is proposed to mediate surface charging, thereby leading to an EDL force that is attractive for the dissimilar system composed of AFM probe and graphene. The results are consistent with the EDL theory, where surface-bound ions (i.e., the ions within the Stern layer) are not able to fully screen the surface charge, which leads to a non-zero potential at the Outer Helmholtz Plane; to compensate this potential, a diffuse layer builds-up. Figure 4C shows an illustration of the proposed EDL for [EMIM][TFSI] on unbiased graphene. Based on the force measurements, the EDL is composed of a structured interfacial region –that we call “extended” Stern layer because it is composed of multiple IL layers- and a diffuse region, where no layers are resolved with the AFM probe. Figure 4C also shows the OHP, but its precise location within the EDL of ILs is unknown. This picture is consistent with previous results for the EDL of other ILs on mica and biased gold^26–31^.

An increase in applied potential is reflected in an increasing onset of the EDL force while the decay length remains constant. An important observation is that the thickness of the IL layers decreases. This is likely because a lesser population of co-ions is present within the extended Stern layer. The trend of the EDL force is reversed at the highest potentials, at which the EDL force becomes less attractive, which suggests that the surface potential is better compensated by the counterions. We propose that this reversal coincides with the transition from overscreening to crowding. The phenomenon of crowding implies that the electric field is high enough to dissociate more effectively the ionic network, so that several layers of counterions populate the interface (Fig. 4D). Thus, counterion crowding compensates better the applied surface potential, which is reflected in a decrease of the effective diffuse potential; this is consistent with the trend observed for the fitted potential (Fig. 4A). Further, an increase in the stored energy should yield a decrease in adhesion (or pull-off force), which agrees well with the reported electrowetting at the highest applied potentials (Fig. 3A).

The shielding effect of graphene in the IL is demonstrated through the remarkably different interfacial structure that is resolved on the selected supports, gold and silica. This is reasonable considering the reported shielding effect of graphene in air^46^ and in water^47^. The different interfacial structure of [EMIM][TFSI] on graphene compared to gold at the same potential must rely on *specific* surface IL-interactions. Image forces, i.e. the electrostatic force on the IL in the neighborhood of a conductor, are expected for both graphene and gold, and hence, they are not expected to be the main source for the observed differences, although image forces are expected to play an important role. First-principle density functional theory (DFT) calculations have shown strong donor-acceptor interactions between ions and graphene^60, 61^ and molecular dynamics simulations suggest π-π interactions to be one of the main mechanisms for adsorption of proteins on graphene^62, 63^. Such π-π interactions are also possible between the π-orbitals in graphene and in the imidazolium ring, and hence, they may be responsible for the enrichment of cations with planar orientation at the graphene interface. Considering that the extended Stern layer at negative potentials appears to be thicker than at positive potentials, it is likely that π- π interactions between graphene and the imidazolium ring of the IL provide more efficient packing of the cation than of the [TFSI]^-^ ion. Similarly, specific interactions between gold and [EMIM][TFSI] could justify the better compensation of the surface potential on gold compared to graphene and consequently, the less pronounced EDL force at all potentials on gold, but this will be investigated in a separate work.

The concept of energy storage by a supercapacitor relies on the storage of charge in a thin EDL, which, according to the classical EDL theory, is expected to be sub-nanometer thickness for an electrolyte at high concentration, after the diffuse layer collapses. Although the electron distribution at the graphene electrode side can strongly influence the interfacial capacitance^64^, it has been demonstrated that the capacitance is dominated by the EDL in the IL if the graphene electrode is composed of more than four graphene sheets. In this case, the potential-dependent differential capacitance^13^ could be roughly estimated with the effective Debye length. Our results show that, for this particular IL, the effective thickness of the EDL on graphene is >1 nm and hence, larger than ideally expected for supercapacitors (<1 nm). Importantly, the measured change of the layer size distribution with potential shown in our force measurements supports that the compacity parameter (or packing efficiency) of [EMIM][TFSI] is not constant but it depends on the applied potential, which is not considered in the simplified EDL theory^13^. The high configurational and conformational entropy of the IL ions, and the ion dissociation as a function of the potential are important parameters to consider for the selection of IL electrolytes for energy storage on graphene electrodes. The observed instability in the force-separation curves (Figure S9) –perhaps due to quasi-crystallization of the adsorbed counterions, as observed on gold^65^- may further decrease the stored energy, and hence, it is an important observation that will drive future studies.

In summary, we have scrutinized the EDL of [EMIM][TFSI] on supported-graphene by measuring surface forces by AFM. The EDL is composed of an extended Stern layer consisting of 4–7 IL layers of different composition, and a diffuse layer, and both respond to the applied potential. On unbiased graphene, significant overscreening occurs due to the preferential adsorption of the imidazolium cations. A transition from overscreening to crowding explains the results at the highest applied potentials. Crowding helps to compensate better the applied potential, which is reflected in a decrease of the EDL force and an increase in electrowetting. Extension of this work to other ILs (e.g. tetraalkylamonium cations) will help to clarify the type of interactions that are responsible for the observed interfacial structure and will contribute to guide the design of IL-based supercapacitors with graphene electrodes.

## Materials and Methods

[EMIM][TFSI] (Iolitec, Alabama, USA) was purchased with > 99% purity. Recent work has shown that for an IL to flow in between graphene sheets, the surface tension of the IL must be in the range 37–45 mN/m, i.e. closely match the surface energy of graphene^66^. No exfoliation of graphene has been observed in [EMIM][TFSI]^67^, which has been attributed to its lower surface tension (33 mN/m), which is also consistent with the stability of our system for at least 12 hours, which was the maximum duration of the experiment.

Graphene was prepared by mechanical exfoliation using Kish graphite, Grade 200 (Graphene Supermarket, New York, USA). Silicon wafers were cleaned in a base piranha solution for 20 minutes at 85 °C, rinsed thoroughly with milliQ water, followed by ethanol rinsing, and dried with dry N_2_. The wafers were then thermally oxidized at 1100 °C for 22 minutes, which led to an oxide layer of ~90 nm that we refer to as silica support or surface. Prior to graphene transfer, the silica surfaces were rinsed with ethanol, dried, and treated in a UV-ozone cleaner for 40 minutes. The gold (Au) working electrode was prepared on the silica support. A chromium (Cr) layer with a thickness of 1 nm and a gold layer with a thickness of 9 nm were thermally evaporated on the silica support with an E-beam evaporator. The Au/Cr film was kept at ~10 nm to yield a semi-transparent film allowing for the fast identification of single- and few-layer graphene with an optical microscope. Prior to exfoliation, the gold electrodes were cleaned for 10 min in a UV-ozone cleaner, followed by thorough rinsing with milliQ water for 1 minute and ethanol for an additional minute. The gold electrodes were then dried with dry N_2_, cleaned by UV-ozone for additional 5 minutes, and rapidly immersed into an ethanol bath for one hour to reduce the gold surface. After reduction, the electrodes were removed from the ethanol bath, and dried with dry N_2_ prior to graphene exfoliation.

Raman spectroscopy and atomic force microscope imaging of the topography were used to detect the number of graphene sheets on silica and gold supports. Raman microspectroscopy measurements were collected with a Nanophoton Raman 11 (Nanophoton, Osaka, Japan) microscope using a 532 nm laser. Laser power was set to at least 1 mW, with exposures lasting at least 15 seconds per spot. The graphene was exposed to ambient laboratory air for ~3–4 hours during identification of suitable graphene samples for experiments, and during Raman spectroscopy. The samples were then stored in vacuum (−0.5 bar) until force measurements were performed, which happened within 24 h to minimize aging.

The atomic force microscopy (AFM) studies were performed on a JPK Nanowizard Ultra (JPK Instruments, Berlin, Germany) under ambient laboratory conditions (T ~ 27 °C, RH ~ 30%). Prior to experiments, all AFM tips were cleaned via UV-Ozone for at least 40 minutes. Graphene images were acquired first in tapping mode in air with a backside gold coated tip (BudgetSensors, Sofia, Bulgaria) with a resonant frequency of ~300 kHz. The images were collected at scan rates between 1–2 Hz. Following imaging in air, the AFM tip was changed to a contact mode tip, and graphene samples and tip were immersed in vacuum-dry [EMIM][TFSI] and the IL was allowed to equilibrate for 40 minutes prior to force measurements. Images of the graphene samples immersed in the IL were typically collected in contact mode with sharp Si-tips (Mikromasch, Tallinn, Estonia) with a spring constant of ~0.5 N/m and a nominal tip radius < 12 nm, at an applied load of 5 nN and scan rates between 1–1.5 Hz. The spring constant was determined according to the thermal noise method^68^.

Force measurements were performed on edge-free areas on single-layer (SLG), few-layer (FLG, 3–4 layers), and multi-layer (MLG) ( > 7) graphene supported on gold and silica, and the control measurements were carried out on silica and gold supports, all in [EMIM][TFSI], in an open cell. Force maps were collected at a constant approach speed of 10 nm/s and an applied normal load that did not exceed 10 nN within scan areas that were at least 500 nm by 500 nm, with at least 64 force isotherms collected per scan area. Although most of the experiments were performed with a Si-tip, some force measurements were conducted with Si_3_N_4_-tips. For the normalization of the force, the tip radius was measured by scanning electron microscopy after the experiment.

The electrochemical AFM cell consists of a gold working electrode and platinum counter and reference electrodes. The volume of the electrochemical cell (~1.3 mL) was filled with the IL to ensure immersion of the counter and reference electrodes. The diameter of the gold working electrode is ~15.6 mm, resulting in a working electrode area of ~1.9 cm^2^. The platinum counter (dia. = 0.5 mm) and reference electrodes (dia. 2 mm) were sonicated in dilute (1 M) HCl for 5 mins, followed by rinsing with milliQ water and ethanol, and dried with dry N_2_.

Water uptake cannot be excluded during the duration of the experiment, but after 12 h it did not exceed 0.45 wt% and 0.1 wt% in the experiments with silica- and gold-supported graphene, respectively, as obtained gravimetrically. The reason for the different water content is the different volume of the open AFM cell used in both experiments (1.3 mL for gold-supported graphene and 20 µL for silica-supported graphene)﻿ and the uptake rate.

### Data availability

The datasets generated and/or analyzed during the current study are available from the corresponding author on reasonable request.

## Electronic supplementary material

Supplementary Material
