# Supplementary material for: Insight into the Electrical Double Layer of an Ionic Liquid on Graphene
Source: Sci Rep. 2017 Jun 26;7:4225. doi: 10.1038/s41598-017-04576-x (PMC5484676; doi:10.1038/s41598-017-04576-x)
Supplement: Supplementary file 1 — Supplementary Material [file 41598_2017_4576_MOESM1_ESM.pdf]

Supplementary Materials for

Insight into the Electrical Double Layer of an Ionic Liquid on Graphene

L. Andres Jurado, Rosa M. Espinosa-Marzal.
University of Illinois at Urbana-Champaign

Correspondence to: rosae@illinois.edu

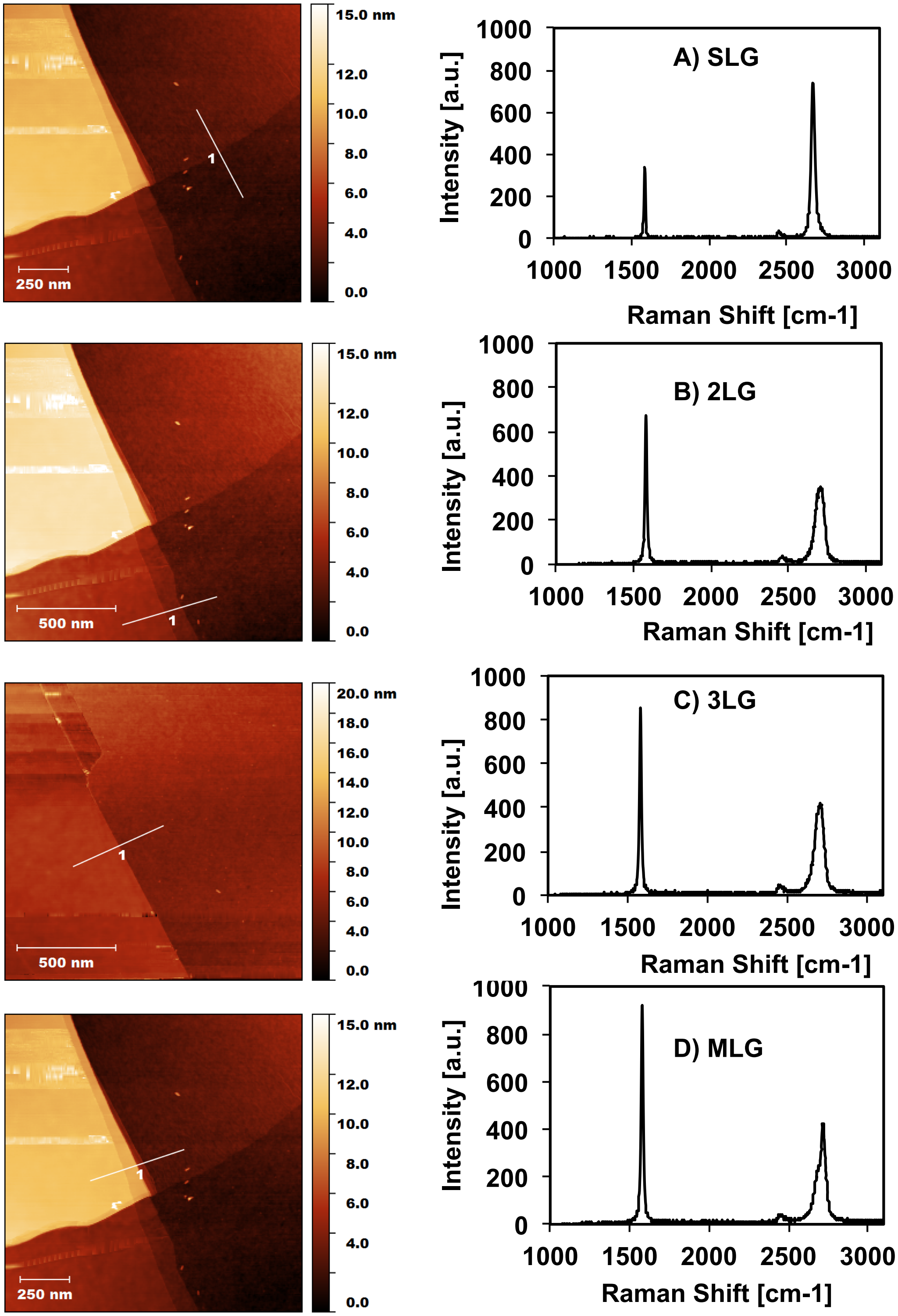

Figure S1: AFM images of the graphene sample immersed in the IL with location of the force maps, all measured with an AFM sharp Si-tip (SLG, 2LG, 3LG and MLG), and corresponding Raman spectra. There is a remarkable decrease in intensity in the 2D band of 2LG compared to SLG, as well as a corresponding Raman shift from ~2630 cm^{-1} to ~2670 cm^{-1}.

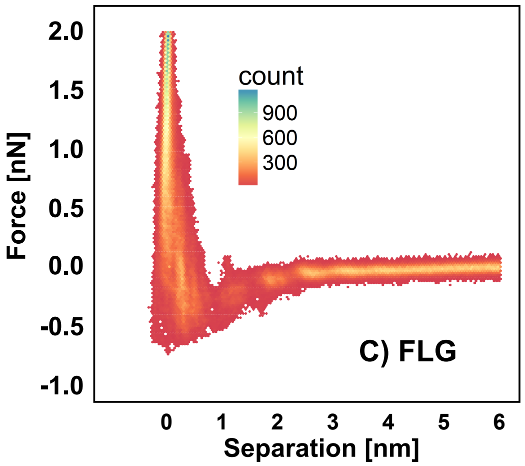

Figure S2: Bivariate histograms for the normal force as a function of the separation (with arbitrary zero at the hard wall) for FLG. The bivariate histograms for the force-separation curves were constructed via hexagonal binning, with a bin size of 150. Red highlights regions of low data density, whereas blue regions highlight regions of high data density. Due to the inherent uncertainty of the absolute tip-substrate separation in AFM force measurements, we note that the abscissa has an arbitrary zero but we label it as "separation".

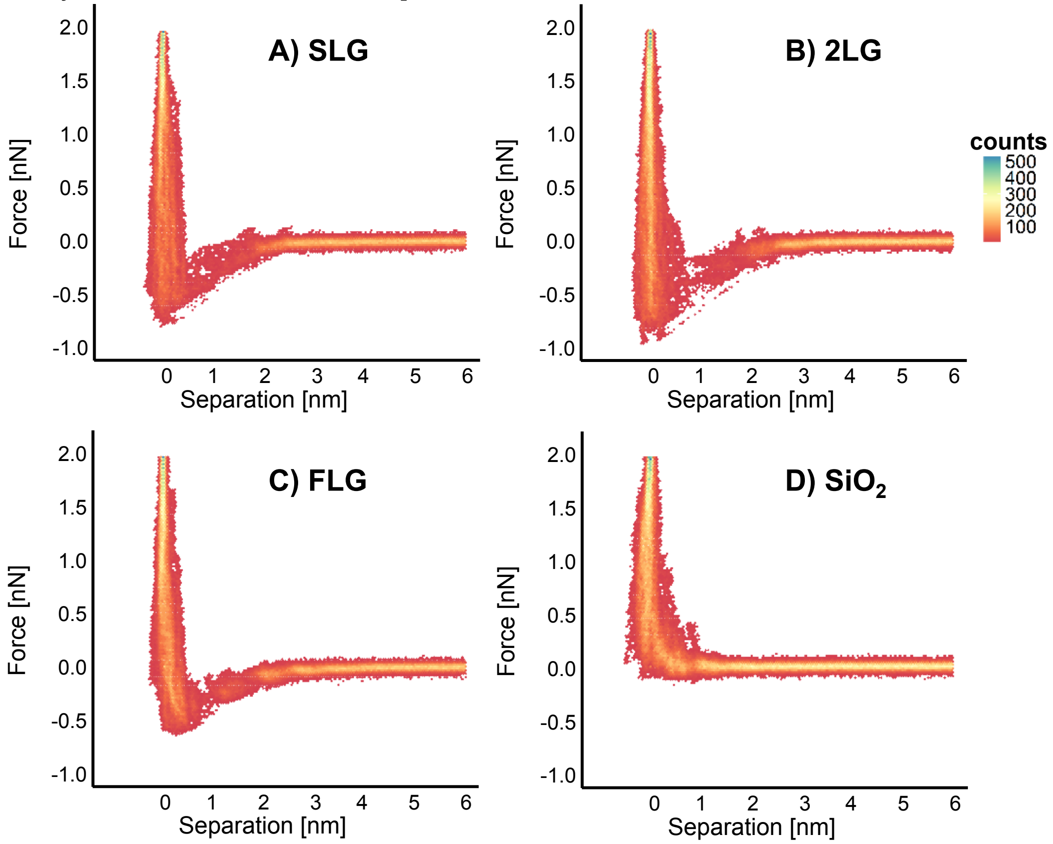

Figure S3: Bivariate histograms for the normal force as a function of the separation (with arbitrary zero at the hard wall) for silica-supported a) SLG, b) 2LG, c) FLG and d) silica. The bivariate histograms were constructed via hexagonal binning, with a bin size of 150. Red highlights regions of low data density, whereas blue regions highlight regions of high data density. The arbitrary zero has been placed at the position of the hard wall.

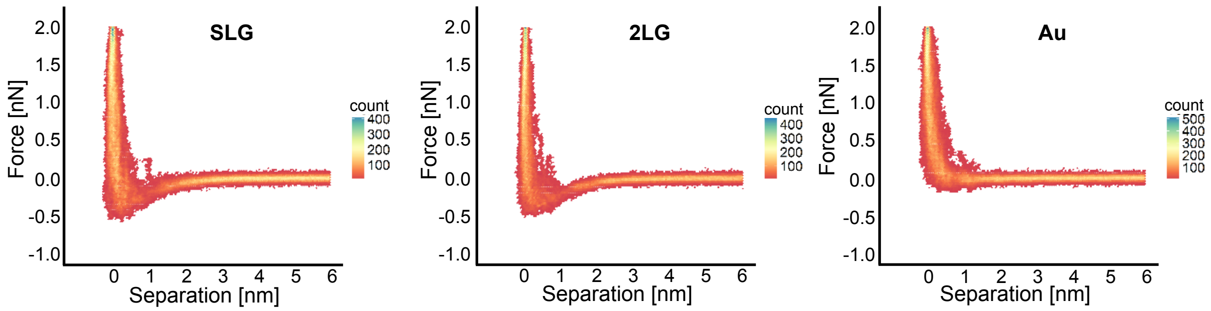

Figure S4: Bivariate histograms for SLG and 2LG on gold-coated substrates resolved with a Si-tip, and on gold as reference. The normal force on SLG and 2LG is attractive similar to the results obtained on silica-supported graphene.

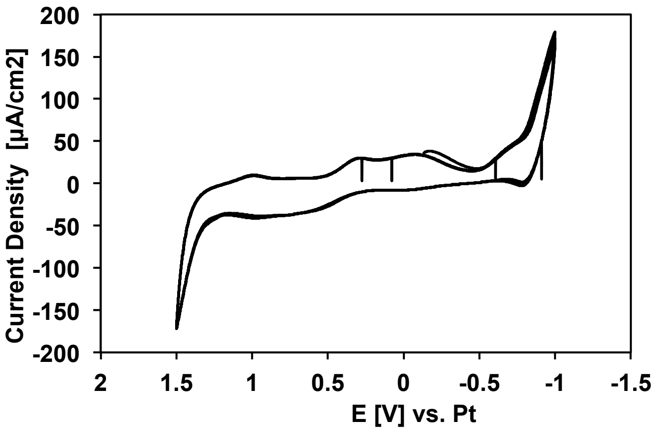

Figure S5: Cyclic voltammetry of [EMIM][TFSI] on the gold-supported graphene electrode at 50 mV s^{-1} for a total of 10 scans between -1.0V to 1.5V. The voltammogram was collected with a CH Instruments 700E Potentiostat.

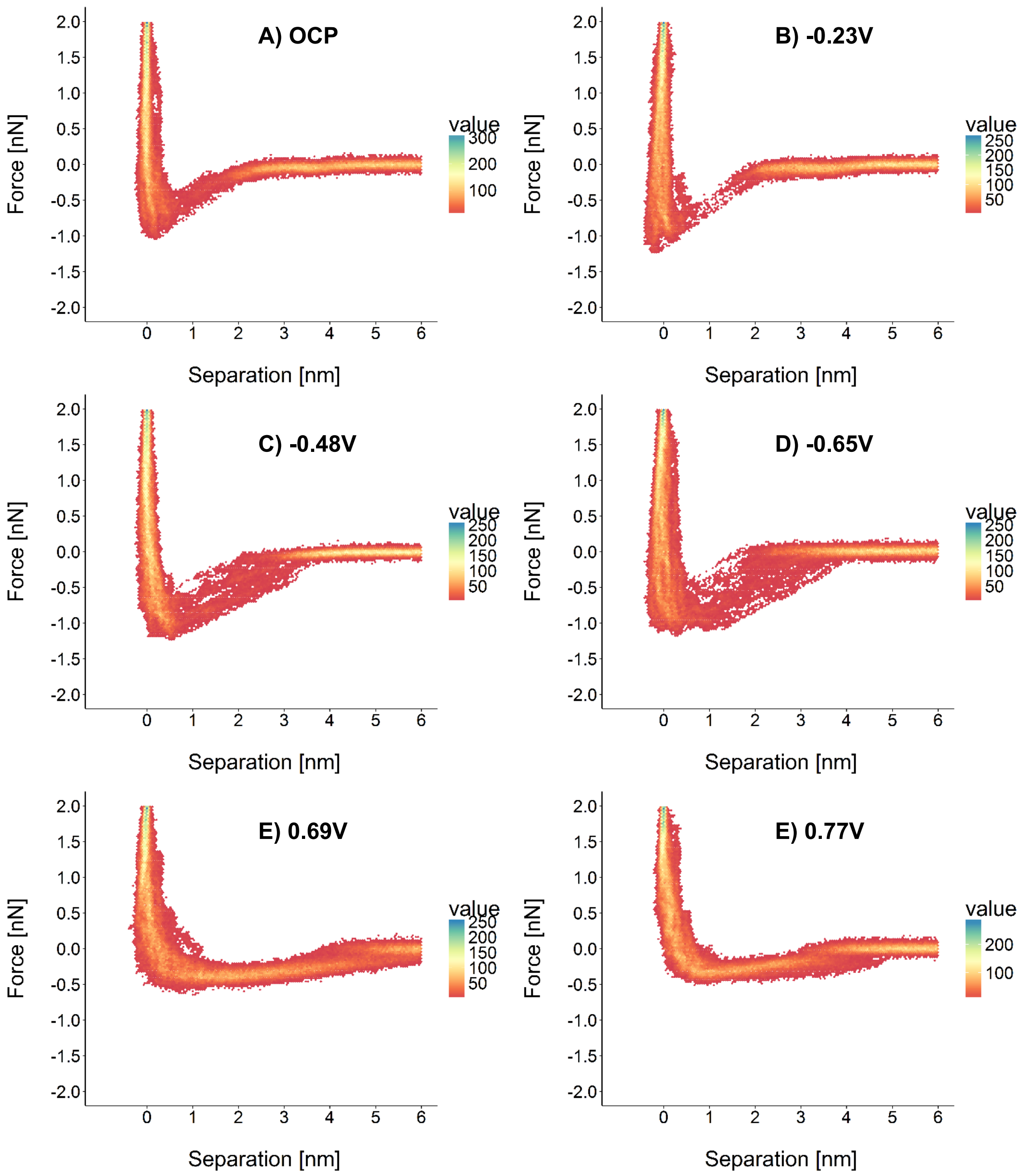

Figure S6: Bivariate histograms for the normal force as a function of the separation (with arbitrary zero at the hard wall) for bilayer graphene as a function of the applied potential measured with a Si-tip. The bivariate histograms were constructed via hexagonal binning, with a bin size of 150. Red highlights regions of low data density, whereas blue regions highlight regions of high data density.

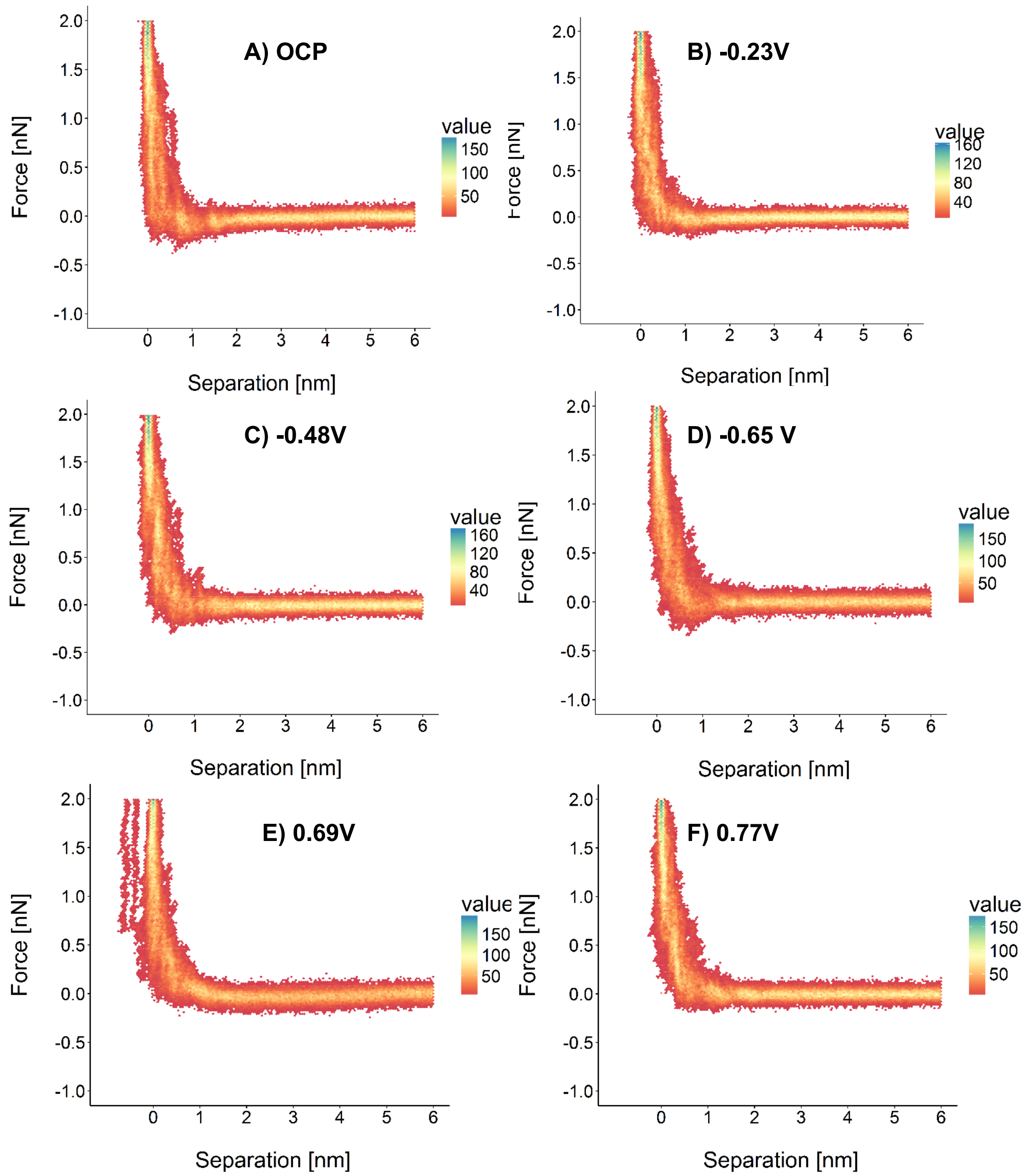

Figure S7: Bivariate histograms for the normal force as a function of the separation (with arbitrary zero at the hard wall) for gold as a function of the applied potential measured with a Si-tip. The bivariate histograms were constructed via hexagonal binning, with a bin size of 150. Red highlights regions of low data density, whereas blue regions highlight regions of high data density.

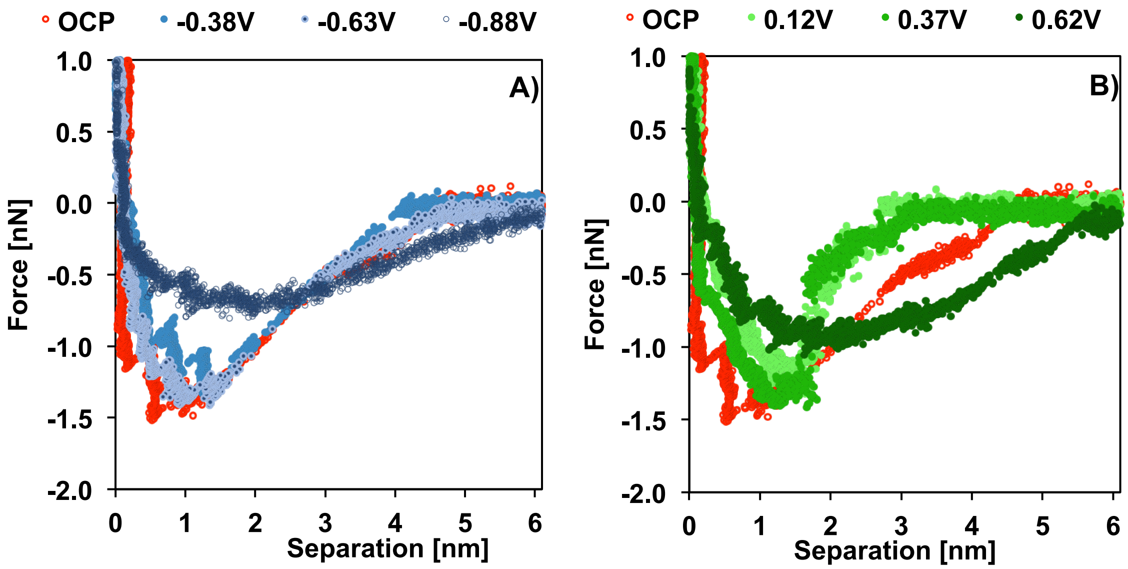

Figure S8: Selected force-separation curves for gold-supported bilayer graphene at varying potentials measured with a Si_3N_4-tip at negative (**A**) and positive (**B**) potentials.

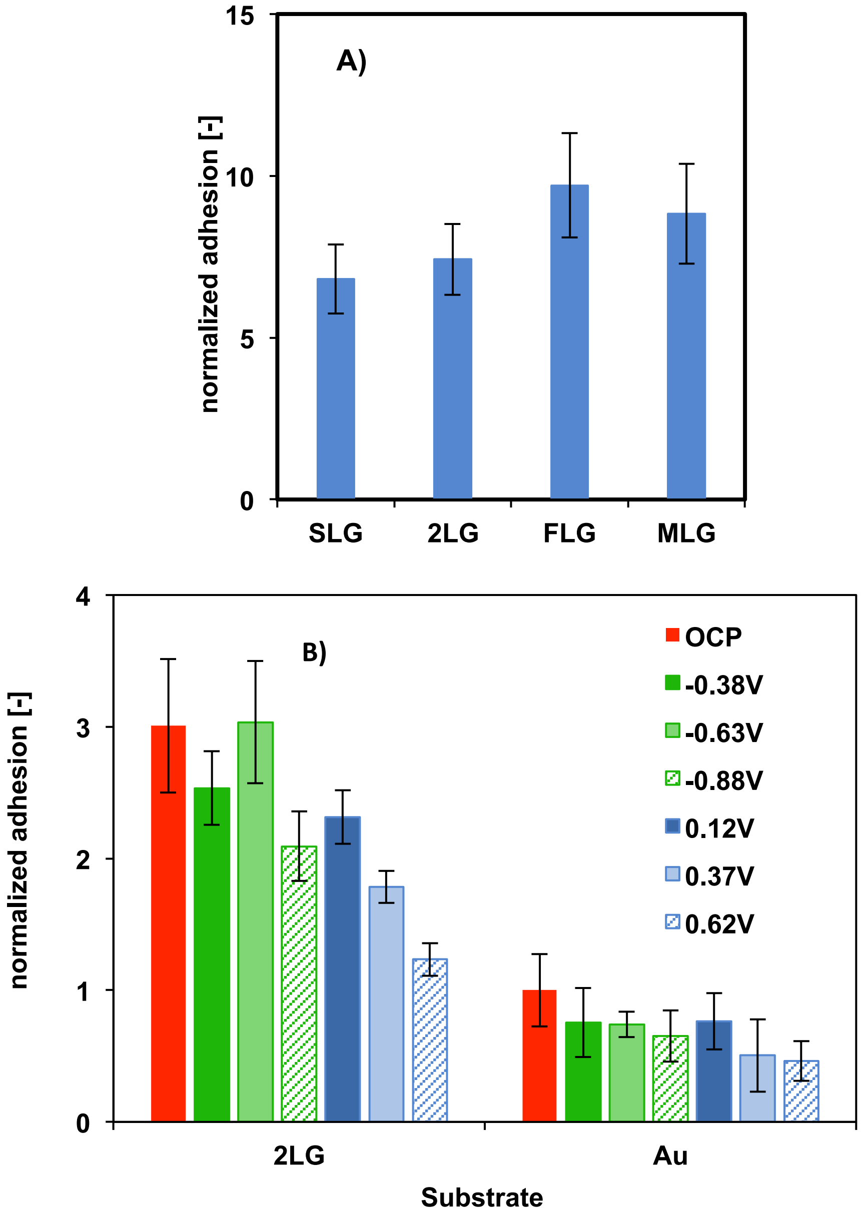

Figure S9: Normalized pull-off force measured on graphene in [EMIM][TFSI]. **A)** Comparison of the normalized adhesion on silica-supported graphene (0.33(0.1) nN) as a function of the number of graphene sheets (for SLG, 2LG, FLG and MLG) measured with a Si-tip. The pull-off force was normalized with respect to the pull-off force on clean silica. **B)** Comparison of the normalized adhesion of 2LG and gold support upon applied potential measured with a Si_3N_4 tip. All values are normalized with respect to the gold substrate at OCP. The applied potentials are shown in the legend. The diagram includes results from different graphene samples prepared on different days but stored 1 day under vacuum after exfoliation and Raman spectroscopy. Similar to the pull-off force in N_2[1], which does not depend on the number of graphene sheets if the contact area remains unchanged, the pull off force did not change remarkably for graphene across many different graphene samples (FLG corresponds to 3 to 4 graphene sheets). This is consistent with a good support of the graphene by the underlying substrate that prevents bending and puckering of graphene sheets.

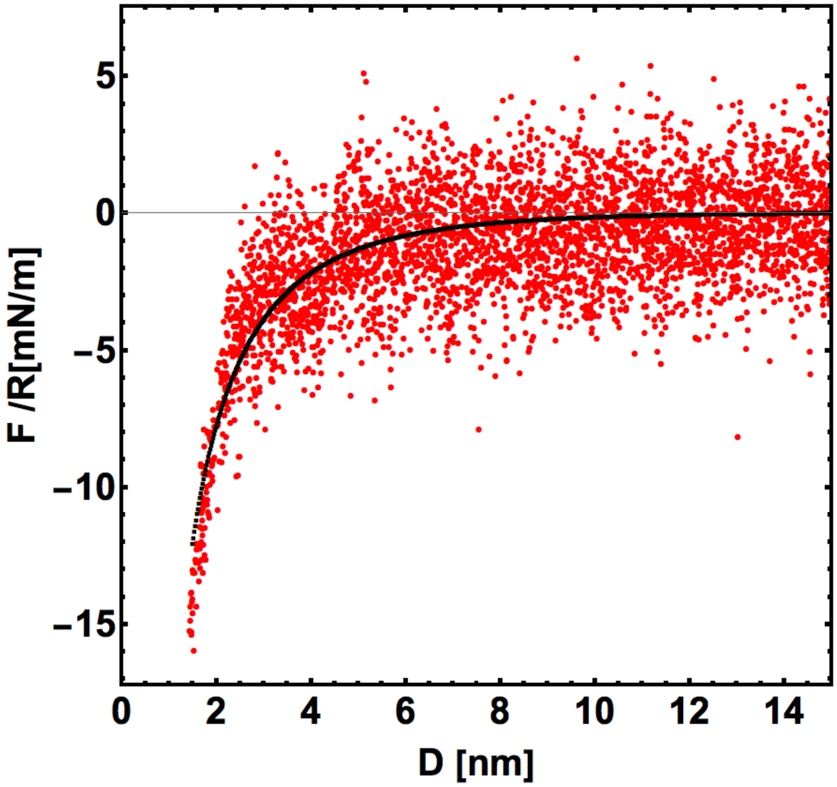

Figure S10: Modeling of the force-separation curves by the DLVO theory for bilayer graphene supported on gold in [EMIM][TFSI] at OCP. The fit gives the surface potential of bilayer graphene (0.196 V), the surface charge of the tip (-0.7 mC/m^2), and the decay length (6 nm). Since non-DLVO forces are neglected, the fit to the DLVO theory is only performed at separations larger than 2 nm with a Hamaker constant= $10.7 \cdot 10^{-20}$ J.

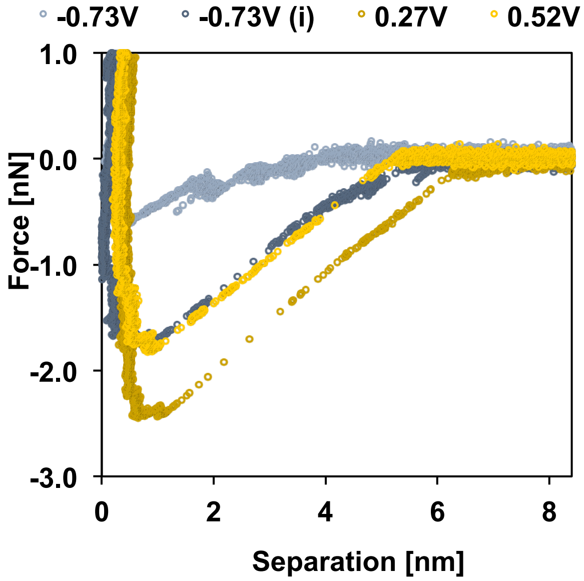

Figure S11: Selected force-separation curves for gold-supported bilayer graphene at varying potentials measured with a Si-tip. The diagram shows force-separation curves in which an instability was detected at the minimum negative potential (compare -0.73 V with -0.73 V(i)) that remained when reversing to positive potentials. Eventually, beyond a positive potential (here 0.52 V), the force became less attractive. Such instability was never observed on gold, and only sometimes on graphene. The instability is also reflected in a significant increase in pull-off force, which suggests that the energy stored within the EDL decreased under these conditions.

MD simulations have shown that an increase in surface potential can bring the interfacial IL structure into a metastable state[2] that could be related to our results. It has been also shown that commensurability between the IL and the surface (gold in the referred work[3]) at high potentials could lead to adsorbed counterions aligned epitaxially along the gold lattice, which could explain quasi-crystallization and superstructures, and thus, that the system remains trapped in a state even upon reversal of the potential.

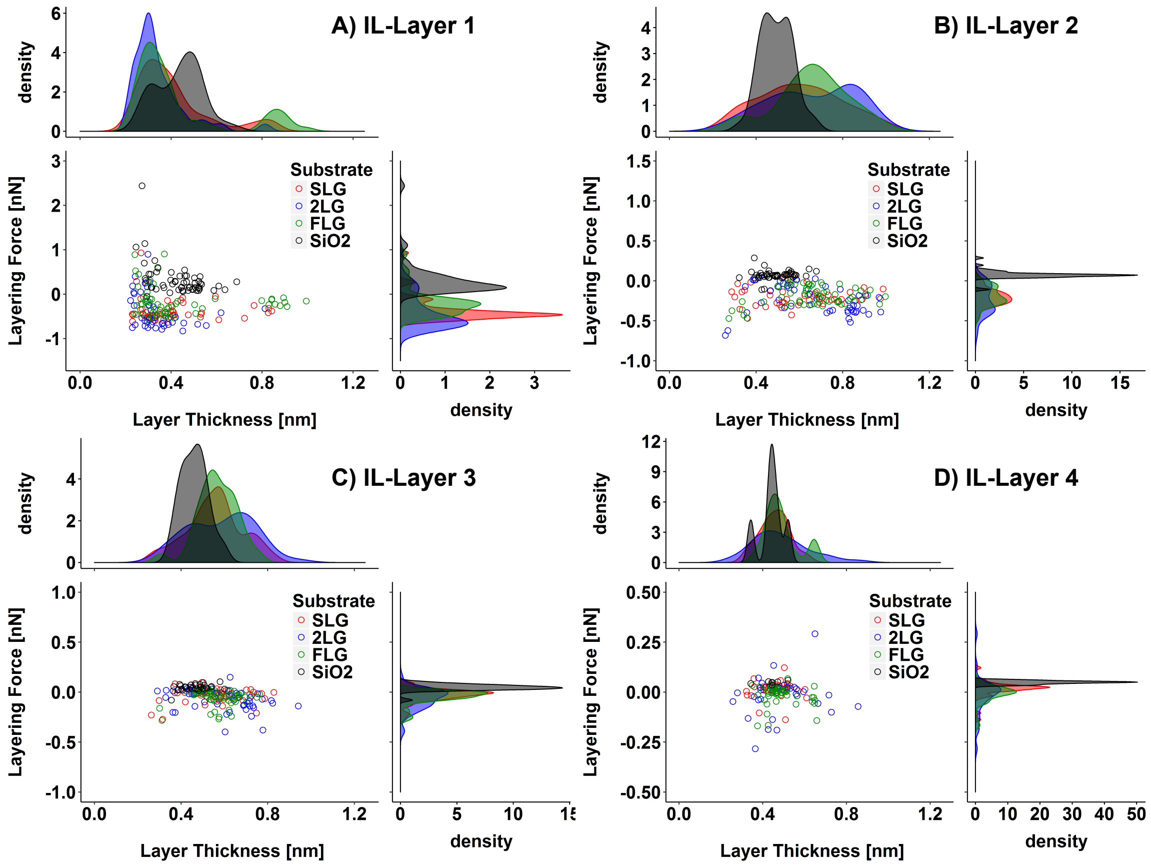

Figure S12: 2D histograms for the IL-layer thicknesses of [EMIM][TFSI] on silica-supported SLG, 2LG and FLG; the results for MLG are similar but are not shown for clarity. The four IL-layers that are closest to the surface are shown, where IL-layer number 1 is the closest to the hard wall, and an increasing index means that the IL-layer is located at an increasing distance from the hard wall. A Si-tip was used to measure the surface forces. The thickness of IL-layer 1 on graphene is ~3.3 Å, as depicted by the red, blue, and green density distribution curves. On silica, the size of IL-layer 1 is ~4.8 Å. An increase in the thickness of IL-layer 2 to ~6-8 Å is observed on graphene, while the silica size remains approximately constant. The thickness of IL-layers 3 and 4 gradually decreases and it eventually reaches the size of the measured IL-layers on silica of ~4.8 Å.

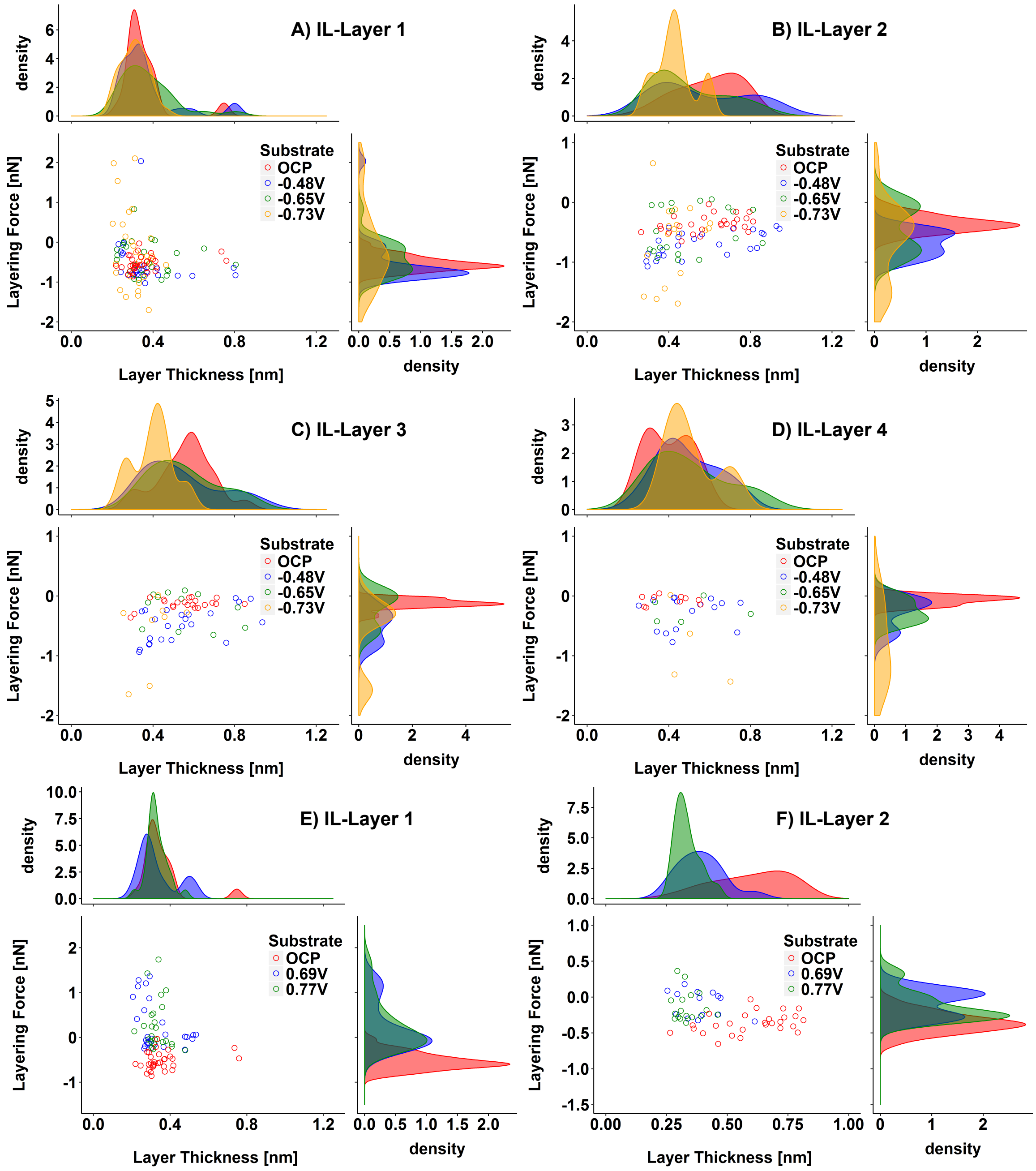

Figure S13: 2D histograms of layering force vs. IL-layer thickness on gold-supported graphene as a function of the potential, measured with a Si-tip. Under negative potentials, the average thickness of IL-layer 1 does not change as a function of the applied potential. IL-layers 2 and 3 decrease in size by increasing the negative or positive potential, in contrast to its expansion at OCP. The thickness of IL-layer 4 at negative potentials is similar to that observed on uncharged graphene, while only three layers at most are detected at positive potentials.

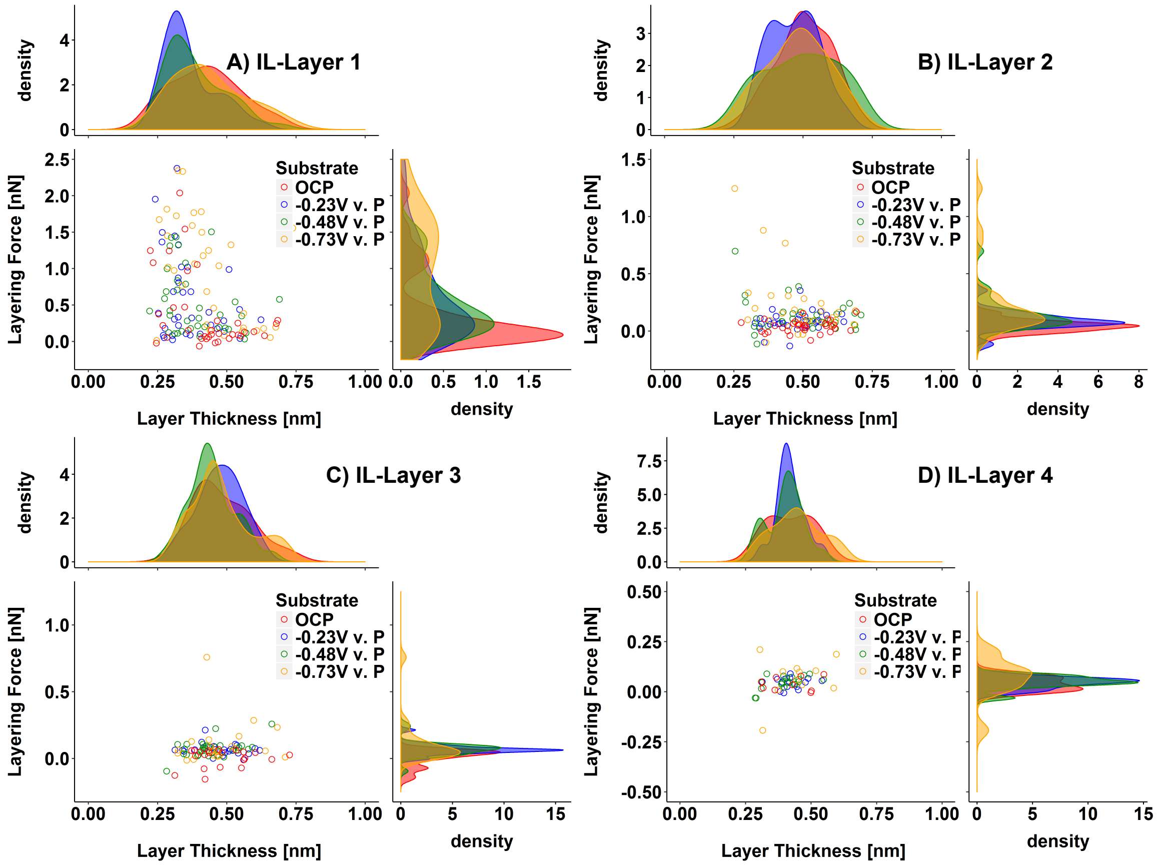

Figure S14: 2D histograms for the layer thickness as a function of the layering force for [EM-IM][TFSI] on gold as a function of the negative potential, measured with a Si-tip.

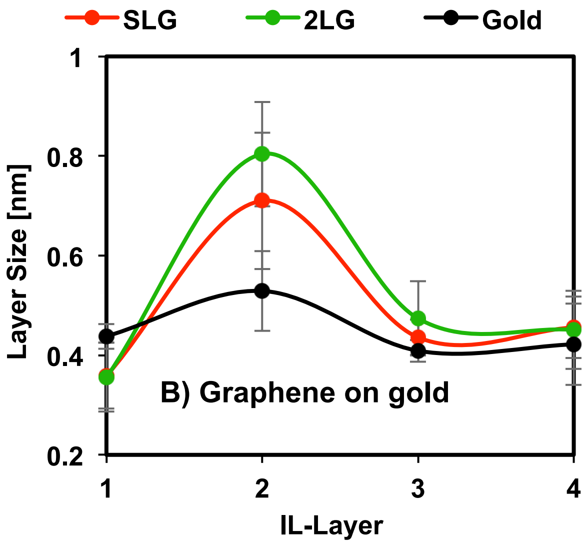

Figure S15: Thickness of the four IL-layers closest to the hard wall on gold-supported SLG and 2LG measured with Si-tip. Although there are deviations between the absolute value of the IL-layers resolved on different graphene samples, as shown here, the thickness of the IL-layer at the hard wall is always the smallest and around ~3.3 Å and the largest thickness is measured for IL-layer 2. The thickness of the IL-layers decreases with distance from the surface.

References:

1 Liu, X.-Z., Li, Q., Egberts, P. & Carpick, R. W. Nanoscale Adhesive Properties of Graphene: The Effect of Sliding History. *Advanced Materials Interfaces* **1**, n/a-n/a, doi:10.1002/admi.201300053 (2014).

2 Burt, R., Birkett, G., Salanne, M. & Zhao, X. S. Molecular Dynamics Simulations of the Influence of Drop Size and Surface Potential on the Contact Angle of Ionic-Liquid Droplets. *J Phys Chem C* **120**, 15244-15250, doi:10.1021/acs.jpcc.6b04696 (2016).

3 Su, Y. Z., Fu, Y. C., Yan, J. W., Chen, Z. B. & Mao, B. W. Double Layer of Au(100)/Ionic Liquid Interface and Its Stability in Imidazolium-Based Ionic Liquids. *Angew Chem Int Edit* **48**, 5148-5151, doi:10.1002/Anie.200900300 (2009).
